# Supplementary material for: High-precision and linear weight updates by subnanosecond pulses in ferroelectric tunnel junction for neuro-inspired computing
Source: Nat Commun. 2022 Feb 4;13:699. doi: 10.1038/s41467-022-28303-x (PMC8816951; doi:10.1038/s41467-022-28303-x)
Supplement: Supplementary file 1 — Supplementary Information [file 41467_2022_28303_MOESM1_ESM.pdf]

Supplementary Information for

High-precision and linear weight updates by subnanosecond pulses in ferroelectric tunnel junction for neuro-inspired computing

Zhen Luo[1,3], Zijian Wang[1,3], Zeyu Guan[1,3], Chao Ma[1], Letian Zhao[1], Chuanchuan Liu[1], Haoyang Sun[1], He Wang[1], Yue Lin[1], Xi Jin[1], Yuewei Yin[1,*] and Xiaoguang Li[1,2,*]

[1]Hefei National Laboratory for Physical Sciences at the Microscale, Department of Physics, and CAS Key Laboratory of Strongly-Coupled Quantum Matter Physics, University of Science and Technology of China, Hefei, China.

[2]Collaborative Innovation Center of Advanced Microstructures, Nanjing University, Nanjing, China.

[3]These authors contributed equally: Zhen Luo, Zijian Wang, Zeyu Guan.

[*]Correspondence and requests for materials should be addressed to X. G. L. (email: lixg@ustc.edu.cn) or to Y. W. Y. (email: yyw@ustc.edu.cn).

List of Contents:

S1. Desirable specifications for synaptic devices

S2. *I-V* curves and transport mechanism analyses

S3. Ferroelectric switching dynamics in (001)-oriented PZT FTJs

S4. Thickness dependence of coercive voltage and resistance for FTJs

S5. FTJs with different top electrodes

S6. Excluding the occurrence of Ag migration

S7. Real-time electric measurements

S8. Resistance switching in FTJs with diameter of 50 nm

S9. Ultrafast resistance switchings by 300 ps pulse voltages

S10. EBSD-SEM and HAADF-STEM did not find grain boundaries in PZT/NSTO

S11. Summary of conductance manipulations of different types of memristors

S12. Neural network simulation for online supervised learning

S13. Ultrafast test circuit with a waveguide

S1. Desirable specifications for synaptic devices

Table S1 Desirable specifications for synaptic devices[1-3]

Performance metrics	Desired targets
Number of conductance states	>100
Conductance dynamic range	>100×
Endurance	$>10^9$ for online learning
Energy consumption	<10 fJ/programming pulse
Nonlinearity	<1 for LTP and >–1 for LTD

Table S1 summarizes the target specifications of artificial synapse devices. For weight updates during the long term potentiation (LTP) and long term depression (LTD) processes, the conductance (*G*) evolution with pulse number (*P*) can be modeled by Equation S1 and S2, respectively[3].

$$G_{\mathrm{LTP}}=C(1-\mathrm{e}^{-\frac{P}{A}})+G_{\min}, \tag{S1}$$

$$G_{\mathrm{LTD}}=D(1-\mathrm{e}^{-\frac{P-P_{\max}}{A}})+G_{\max}, \tag{S2}$$

$$\alpha=1.726/(A+0.162). \tag{S3}$$

Here, $G_{\max}$ and $G_{\min}$ are the maximum and minimum conductances of the device, respectively. $P_{\max}$ is the maximum number of pulses to tune the conductance. Parameter α is the nonlinearity of weight updates. By fitting the conductance *versus* pulse number with Equation S1 or S2, the parameter A can be obtained, and α is then calculated by Equation S3. The lower α is, the more linear the curve is.

S2. *I-V* curves and transport mechanism analyses

Fig. S1a shows a typical *I-V* hysteresis loop of the Ag/$PbZr_{0.52}Ti_{0.48}O_3$ (PZT)/Nb:$SrTiO_3$ (NSTO) FTJ, indicating its memristive characteristic, similar to the earlier report[4]. In addition, the *I-V* curve clearly shows a rectifying transport character, suggesting the existence of the Schottky barrier for the MFS-type FTJs. To explore the transport and resistive switching mechanisms, the *I-V* curves at different temperatures from 150 to 270 K were measured at ON and OFF states of the FTJ with (111)-oriented PZT barrier, as shown in Fig. S1b and c, respectively. To semi-quantitatively analyze the ferroelectricity tuned barrier, the thermally assisted tunneling model can be used after simplifying the composite

barrier to be a Schottky barrier. The thermally-assisted tunneling current can be described by[5,6]

$$J_F = J_S(T)\exp(qV/E_0). \quad \text{(S4)}$$

Through fitting the $\ln J_F$ *vs.* V curves using Equation S4, the J_S and $1/E_0$ at different temperatures can be extracted, and their relationship follows Equation S5:

$$J_S = \frac{A^* T^2 \pi^{1/2} E_{00}^{1/2} q^{1/2}[(V_{bi}-V)+\phi_n]^{1/2}}{k_B T \cosh(E_{00}/k_B T)} \times \exp\left[q\left(\frac{\phi_n}{k_B T} - \frac{V_{bi}+\phi_n}{E_0}\right)\right], \quad \text{(S5)}$$

where $E_0 = nk_BT$ and $E_{00} = \frac{qh}{4\pi}\left[N_D/m_n^*\varepsilon_r\varepsilon_0\right]^{1/2}$. The temperature dependent relative permittivity $\varepsilon_r(T)$ of NSTO can be described by Barrett's formula[7,8]:

$$\varepsilon_r(T) = 1635/[\coth(44.1/T) - 0.937]. \quad \text{(S6)}$$

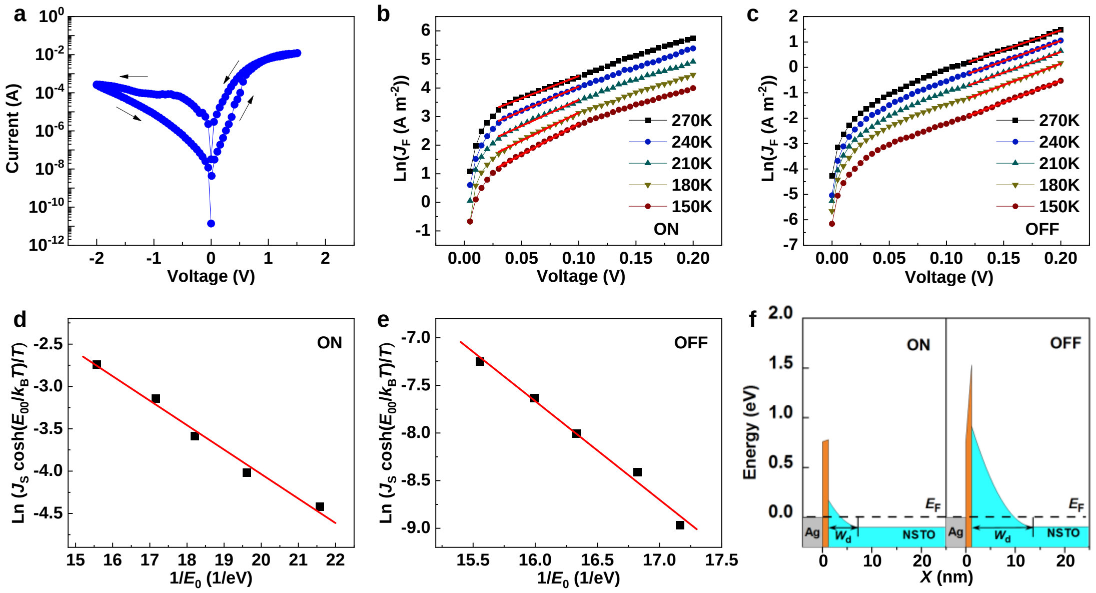

Supplementary Fig. S1 *I-V* curves and transport mechanism analyses of the Ag/PZT/NSTO FTJ. a *I-V* curves measured by sweeping the voltage from 0 to 2 V, then 2 V to −2 V, and finally back to 0 V. The arrows indicate the voltage sweeping direction. **b, c** $\ln J_F$ *vs.* voltage at various temperatures from 270 to 150 K at ON and OFF states, respectively. **d, e** $\ln[J_S\cosh(E_{00}/k_BT)/T]$ *vs.* $1/E_0$ plots for ON and OFF states, respectively. The red solid lines are linear fitting results. **f** Energy profiles of ON and OFF states of Ag/PZT/NSTO FTJs by considering the work function of Ag (~4.26 eV), and electron affinities of PZT (~3.5 eV) and NSTO (~4.0 eV)[9,10].

Based on the model, the Schottky barrier height V_{bi} can be extracted from the slope of the linear fitting of $\ln[J_S\cosh(E_{00}/k_BT)/T]$ on $1/E_0$, as shown in Supplementary Fig. S1d and e. Besides, the width W_d of the depletion layer can be estimated from $W_d = (2\varepsilon_0\varepsilon_r V_{bi}/qN_D)^{1/2}$, where the calculated ε_r of NSTO is about 280 at room temperature

according to the Supplementary Equation S6. As shown in Fig. S1f, when the FTJ switches to ON state (high conductance), the values of V_{bi} and W_d are about 0.28 eV and 6.6 nm, respectively. While for OFF state (low conductance), the V_{bi} and W_d are about 1.03 eV and 12.6 nm, respectively.

S3. Ferroelectric switching dynamics in (001)-oriented PZT FTJs

The ferroelectric switching dynamics of FTJs with (001)-oriented PZT were also investigated as a comparison with that of (111)-oriented PZT. Obviously, there is no plateau during switching, as shown in Supplementary Fig. S2, which implies that only one step switching process occurs in FTJs with (001)-oriented PZT.

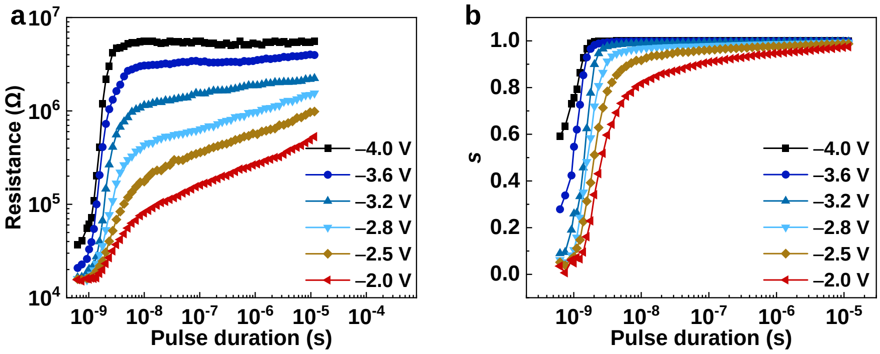

Supplementary Fig. S2 Ferroelectric switching dynamics in (001)-oriented PZT FTJs. **a** Resistances measured at 0.05 V *versus* pulse duration for the FTJ with (001)-oriented PZT. **b** Relative area fraction of the ferroelectric up domain *versus* pulse duration for the FTJ with (001)-oriented PZT.

S4. Thickness dependence of coercive voltage and resistance for FTJs

To figure out the relationship between coercive voltage (V_c) of FTJ and thickness (d) of ferroelectric film, the resistance *vs*. pulsed voltage loops of FTJs with different PZT thicknesses and pulse durations (t_d = 10 ns and 630 ps) were measured, as shown in Fig. S3a and b. The coercive voltages V_c can be calculated through averaging the absolute values of positive and negative coercive voltages in Fig. S3a and b. The values of V_c decrease with decreasing PZT thickness d from 6.0 nm to 1.2 nm, and the relationship between V_c and d can be fitted by a power law:

$$V_c \propto d^{\alpha}. \qquad \text{(S7)}$$

The slopes α are 0.30 for t_d = 10 ns and 0.26 for t_d = 630 ps, respectively, as shown in Fig. S3c and d. This is very close to the Kay-Dunn law $V_c \propto d^{1/3}$ for ferroelectric thin films[11], such as α = 0.38 for PZT[12] and α = 0.331 for $BiFeO_3$[13]. The slightly smaller α of our FTJ

than the slope parameter of Kay-Dunn law[11] may be related to the depleted region at the semiconductor interface which shares the applied voltage.

Besides, it is noted that with increasing PZT thickness, the FTJ resistance increases as a result. As shown in Fig. S3e and f, the extracted resistances for both ON and OFF states from Fig. S3a and b clearly show exponential dependences on *d*, which indicates the tunneling effect in FTJs[14].

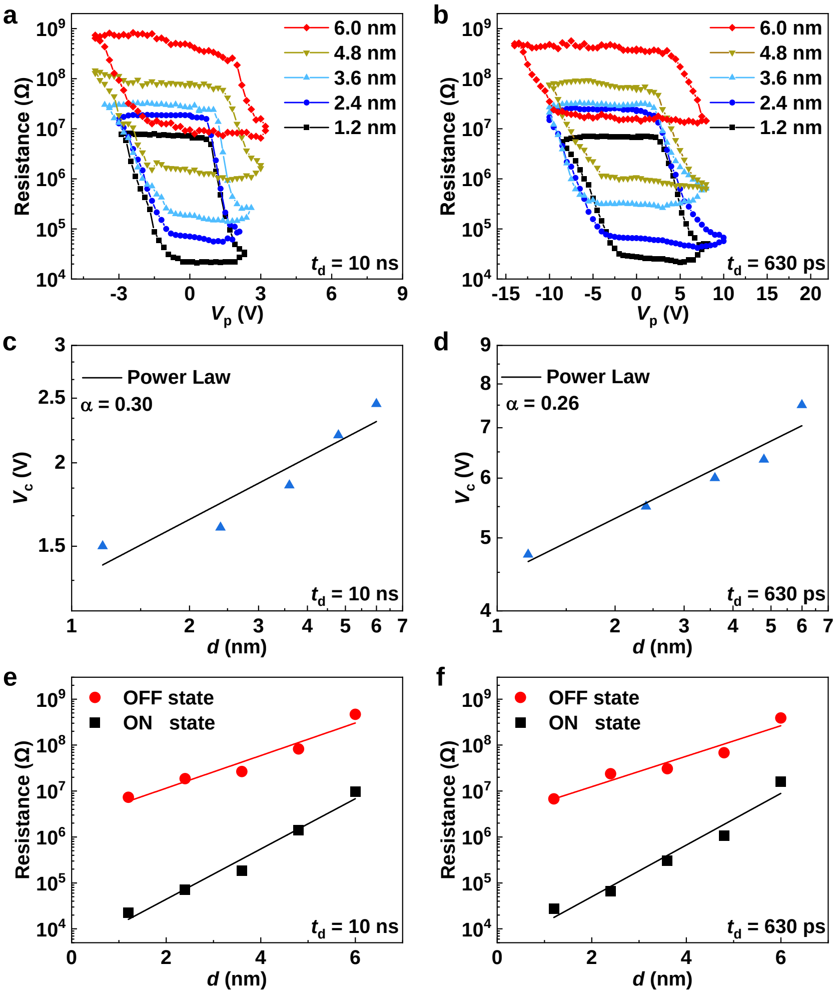

Supplementary Fig. S3 Ferroelectric film thickness dependence of coercive voltage and resistance for FTJs. a, b Resistance *vs.* pulsed voltage loops with pulse durations of t_d = 10 ns and 630 ps, respectively. **c, d** Thickness dependent coercive voltages with t_d = 10 ns and 630 ps, respectively. The solid lines are the fitting results by using a power law $V_c \propto d^{\alpha}$. **e, f** Thickness (*d*) dependent resistances of ON and OFF states with t_d = 10 ns and 630 ps, respectively. The solid lines are the fitting results by an exponential law.

S5. FTJs with different top electrodes

Fig. S4 shows resistance *vs.* pulsed voltage loops for the FTJs with different electrodes, including Pt, Cu, and Ag. The resistance switching characteristics of the FTJs with the

different electrodes are listed in Table S2. With increasing work functions of electrodes from Ag to Pt, the ON/OFF ratio increases, while the coercive voltages increase obviously. Considering that the ON/OFF ratio of >100 for the FTJ with Ag electrode is sufficient to meet the target performance for an synaptic device (Table S1)[15], Ag electrode was then chosen for reducing the operation voltage and enhancing the operation speed. Besides, it is noted that the FTJ with CMOS-compatible Cu electrode can also work at subnanosecond but with a higher voltage.

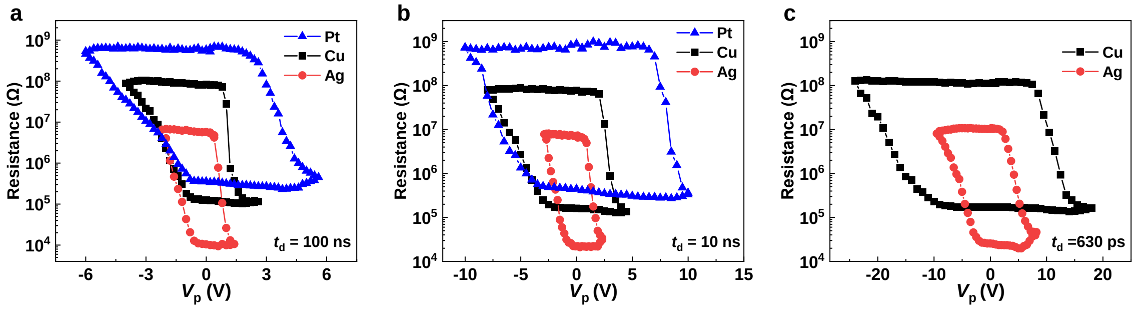

Supplementary Fig. S4 Resistance *vs.* pulsed voltage loops of FTJs with different top electrodes. a t_d = 100 ns, **b** t_d = 10 ns, and **c** t_d = 630 ps.

Table S2 Resistance switching characters of FTJs with different electrodes

Top electrode	Pt	Cu	Ag
Work function	5.65 eV	4.5 eV	4.26 eV
Coercive voltage (t_d = 100 ns)	3.6 V/–3 V	1.1 V/–2 V	0.7 V /–1.4 V
Coercive voltage (t_d = 10 ns)	8.2 V/–6.7V	2.7 V/–5 V	1.3 V/–1.7 V
Coercive voltage (t_d = 630 ps)	–	11.5 V/–17 V	4.7 V/–5 V
ON/OFF ratio	3000	1000	500

In addition, the electrodes also influence switching endurance of FTJ. Fig. S5 shows the switching endurances of FTJs with Pt and Ag electrodes using the same pulse duration t_d = 1 μs. For the FTJ with Pt top electrode, larger operation voltages of 3.5 V/–4.5 V are required to realize the ON/OFF ratio ~1500 and the corresponding switching endurance is ~2×10^6. By decreasing applied voltages to 2.7 V/–3.2 V, the ON/OFF ratio decreases to ~200, as shown in Fig. S5b, and the corresponding switching endurance increases to ~10^8. While for FTJ with Ag electrode with an ON/OFF ratio of ~200, the switching endurance is as high as 6×10^8. This should be due to the lower operation voltages of 1.5 V/–2.5 V for the FTJ with Ag electrode.

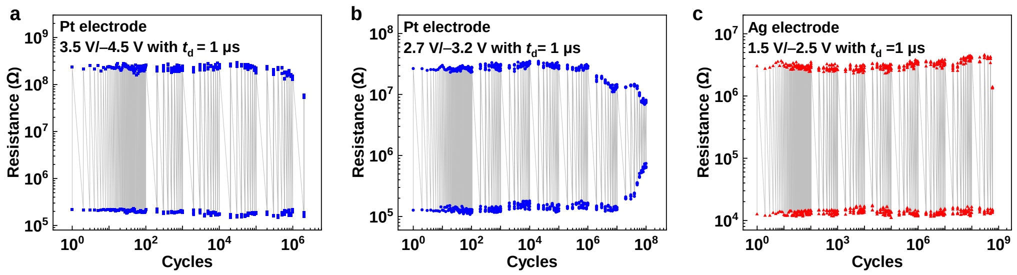

Supplementary Fig. S5 Switching endurances of FTJs with Pt and Ag top electrodes. **a, b** Endurance of Pt electrode FTJ switched by applying 3.5 V/−4.5 V and 2.7 V/−3.2 V pulsed voltages, respectively. **c** Endurance of Ag electrode FTJ by applying 1.5 V/−2.5 V voltage pulses. The pulse durations t_d in **a, b,** and **c** are 1 μs.

S6. Excluding the occurrence of Ag migration.

The resistance switching mechanism of our FTJ is closely related to the ferroelectricity-affected band structure instead of Ag migration. There are some experimental evidences to exclude the occurrence of Ag migration.

1) The resistance switching characteristics based on the tunneling across a ferroelectric barrier of FTJs are different from these of Ag migration. For Ag conducting filament based memristors, the current increases abruptly when Ag filament formed[16]. Thus, the *I*-*V* curve is typically linear at ON state, and the current would decrease with increasing temperature because the conduction mechanism follows a metallic behavior[17]. While for our FTJs, as shown in Fig. S1, the *I*-*V* curves at ON state follow a thermally-assisted tunneling model, suggesting a quantum tunneling mechanism. In addition, as shown in Fig. S3, both ON and OFF states of resistances show exponential dependences on the PZT thickness, also indicating a tunneling effect in FTJs[14].

2) As shown in Fig. S4, the resistance switching behavior of the FTJ with Ag electrode is similar to these with Cu and Pt electrodes, supporting the same underlying resistance switching mechanism among different electrodes[18]. This is also an evidence to exclude the occurrence of Ag filaments in the samples.

3) The resistive switching of the Ag/BTO/NSTO FTJ is closely correlated with a nucleation-limited-switching (NLS) model of the ferroelectric domain dynamics[19], as demonstrated in Fig. 3 of the manuscript.

All the above experimental results confirm that the resistance switching of the Ag/BTO/NSTO FTJ is caused by ferroelectric polarization switching rather than the conduction bridge based on Ag filaments.

S7. Real-time electrical measurements

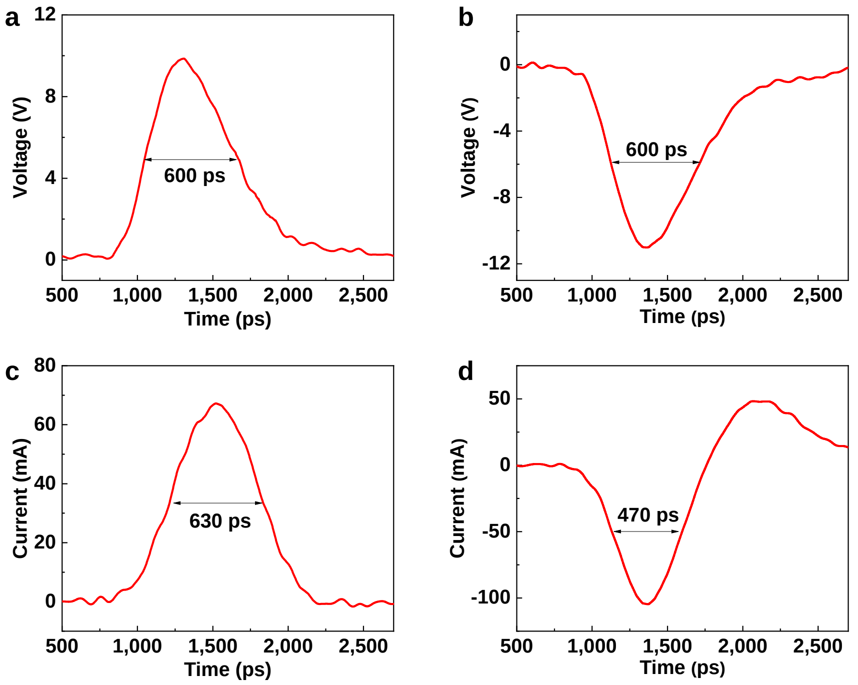

Supplementary Fig. S6 Real-time electrical measurement for Ag/PZT(111)/NSTO FTJ. a, b Voltage pulses of 10 V and −11 V in amplitudes with 600 ps in durations applied to the FTJ top electrode, respectively. **c**, **d** Signal transmitted through the FTJ with durations of 630 ps and 470 ps corresponding to 10 V and −11V applied voltage pulses, respectively.

To ensure that sub-nanosecond pulsed voltages are successfully delivered to the FTJ and to measure the current through the FTJ for energy consumption estimation, we conducted a real-time electrical measurement that is similar to the previous literatures[20,21]. By applying positive and negative voltage pulses of ~600 ps shown in Fig. S6a and b, respectively, the transmitted current signals through the FTJ are captured by the oscilloscope, as depicted in Fig. S6c and d, respectively. It can be seen that the transmitted positive pulse is extended from 600 ps to 630 ps (defined as full width at half maximum), while the negative signal is deformed in shape with an even smaller pulse width ~470 ps. The different deformed current signals between positive and negative voltage pulses are related to the rectification characteristics of our FTJ (see Fig. S1), and this will lead to the different impedance mismatching conditions. Similar deformed transmitted signals have also been reported in other memristors[22,23]. In addition, under the positive and negative pulses, the write current densities can be estimated to be about 1.0×10^3 and 1.3×10^3 A/cm^2, respectively, and these lead to the energy consumptions per operating pulse ~440 and ~520 pJ, respectively.

S8. Resistance switching in FTJs with diameter of 50 nm

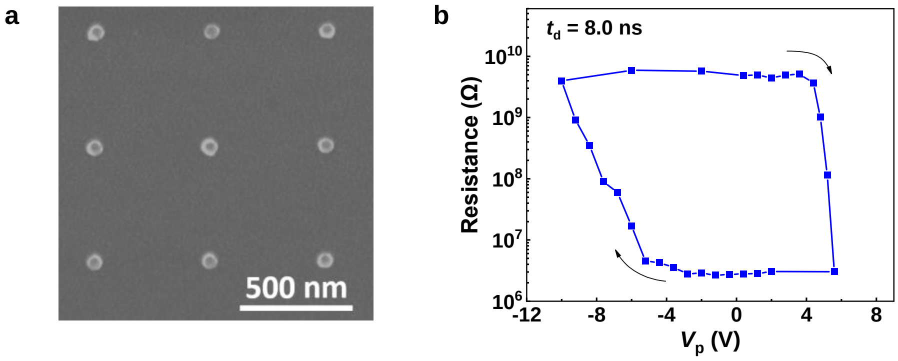

Supplementary Fig. S7 Resistance switching in FTJs with diameter of 50 nm. **a** SEM image, and **b** R-V_p loop result with t_d = 8 ns. The arrows indicate the voltage sweeping direction.

To further reduce the energy consumption, the nanoscale Pt/PZT/NSTO devices with top electrode diameter of ~50 nm were prepared, as shown in Fig. S7. The corresponding R-V_p loop (t_d = 8 ns) measured through a conductive tip is shown in Fig. S7b. The multi-state can be achieved through a low resistance state switching to a high resistance state, and the ON/OFF ratio is about 2×10^3. The switching current density is ~7.6×10^3 A/cm^2 under 4.4 V. The write energy per bit for the 50 nm diameter FTJ is about 5.3 fJ, which shows ultralow energy consumption compared with the phase change memristors and ferromagnetic tunnel junction memristors[24,25].

S9. Ultrafast resistance switchings by 300 ps pulsed voltages

By increasing the amplitude of voltage pulses, the resistive switching speed can be further accelerated to 300 ps, which is the highest resistance switching speed among FTJs[6,19,21]. As shown in Fig. S8, the distinguishable multi-state resistances were obtained by 300 ps pulsed voltages. The real-time measurement was also carried out, as shown in Fig. S8a. The pulsed signal transmitted cross the device with a duration of 300 ps was monitored by an oscilloscope. As shown in Fig. S8b, the resistance can be tuned continuously to various intermediate resistances by varying the negative maximum voltage V_p^{max-} from –6.7 V to –13.3 V. Fig. S8c shows that different resistance states can be achieved. An ON/OFF ratio of >10 can be realized by voltages of 12.7 V/–12.7 V with t_d = 300 ps. Although the operation voltages are larger than typical primary power voltage on MOS devices[26], it is lower than the operation voltage of NAND flash which is usually around 20 V[27]. Therefore, similar to the NAND flash, a charge pump circuit may be used to realize affordable operation voltages for ultrafast FTJs[28].

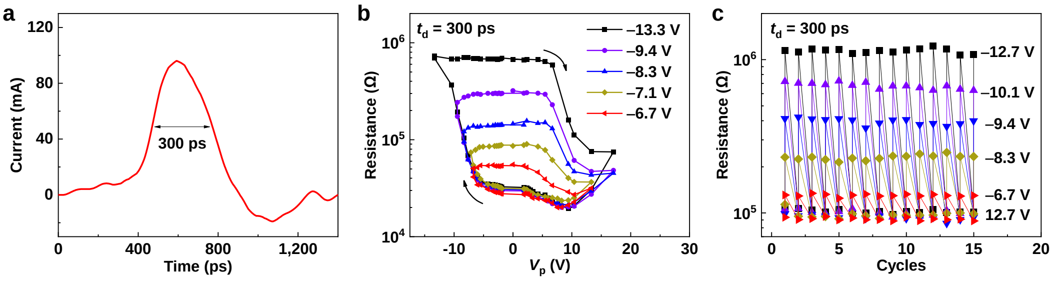

Supplementary Fig. S8 Ultrafast resistance switchings by 300 ps pulsed voltages. a Real-time current waveform through the FTJ. **b** *R*-V_p loops and **c** resistance switchings among different states by different V_p with t_d = 300 ps. The arrows in **b** indicate the sweeping direction of V_p.

S10. EBSD-SEM and HAADF-STEM did not find grain boundaries in PZT/NSTO

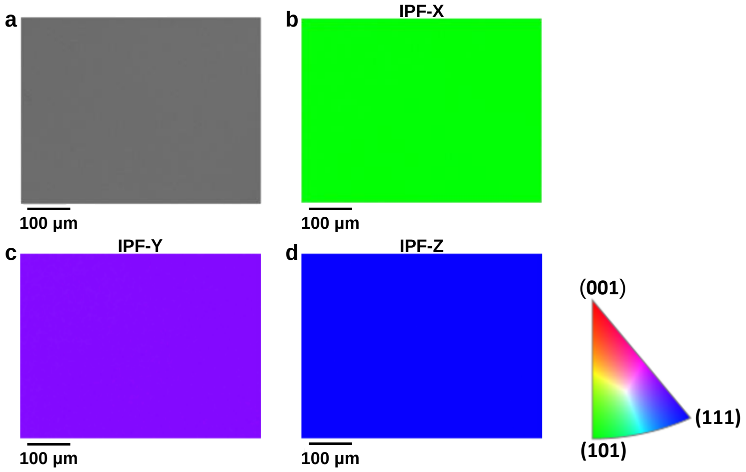

Supplementary Fig. S9 Electron backscattered diffraction (EBSD) maps of PZT/NSTO. a Scanning electron microscopy (SEM) image corresponding to the following EBSD maps. **b, c,** and **d** electron back scattered diffraction (EBSD) inverse pole figure (IPF) maps: IPF-X, IPF-Y, IPF-Z of NSTO substrate, respectively.

It is known that the grain boundary may reduce the device endurance, because oxygen vacancies could accumulate at grain boundaries, pin the ferroelectric domain and degrade the insulation of devices[29,30]. For our FTJ device, the ultrathin ~1.2 nm PZT ferroelectric film is epitaxially grown on the (111)-oriented NSTO single crystal substrate, and the orientation of film is consistent with the substrate. As shown in Fig. S9, the uniform orientation of the PZT/NSTO is revealed by the electron back scattered diffraction (EBSD) inverse pole figure (IPF) maps of 500×500 μm^2, indicating its monocrystal nature. To further investigate the possible grain boundaries in PZT film, the high-angle annular dark-field scanning transmission electron microscopy (HAADF-STEM) was measured at the

cross-section of a PZT/NSTO sample. The STEM sample was fabricated by focused ion beam and its lateral size is ~ 1.5 μm. This 1.5 μm STEM sample was detected from one side to the other by measuring local HAADF-STEM images, and 5 representative pictures are shown in Fig. S10. No grain boundary was observed in the tested area. However, this cannot entirely exclude the possibility that there exist a few grain boundaries which will be detrimental for the device endurance. Improving sample qualities by reducing grain boundaries is always important.

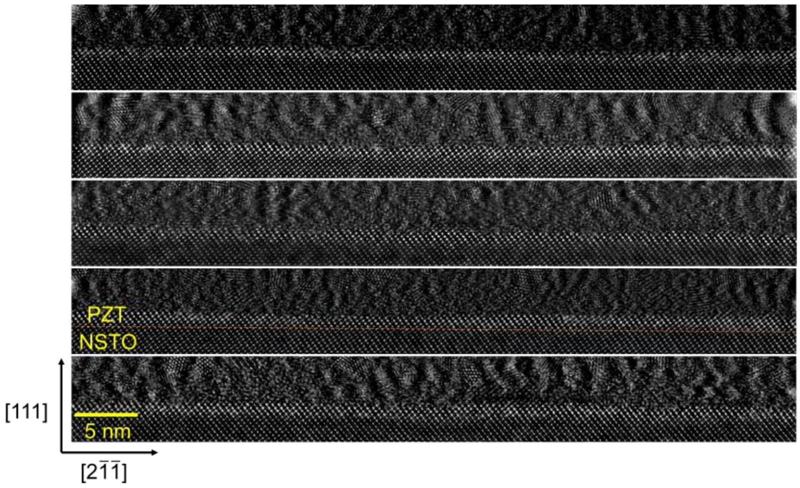

Supplementary Fig. S10 Representative HAADF-STEM images of the PZT/NSTO heterostructure. 5 representative HAADF-STEM images of (111)-oriented PZT (1.2 nm)/NSTO.

S11. Summary of conductance manipulations of different types of memristors

The representative performances of different types of memristors including conducting filament modulated memristors, interfacial ionic displacement type memristors, ferroelectric field effect transistor (FeFET) memristors, and FTJ memristors are listed in Table S3. It is noted that the conductance manipulations operated by variable voltages are usually more linear than using invariable voltage. Thus, variable voltage scheme is chosen for manipulating conductance. Based on the continuous ferroelectric switching dynamics of (111)-oriented PZT (~1.2 nm) of FTJs, the conductance manipulation of our device is very linear (nonlinearity ~1), satisfying the targeted specification for a synaptic device[15]. Besides the outstanding linearity, our device shows comprehensive performance advantages with high-precision (256 states, 8 bits), reproducible (cycle-to-cycle variation ~2.06%), symmetric weight updates and subnanosecond operation speed.

Table S3 Overview of representative performances of different types of memristors[31-37]

Metrics / Device Type	Mechanism	Nonlinearity (Set/Reset)	Dynamic range	Cycle variation	State numbers	Operation voltage
Ta/HfO_x/Pt [31]	Filament modulation	0.1 / –0.1	10	2%	100	0.6 ~1.6 V@ 10 μs
TEL/HfO_x [32]	Filament modulation	0.96/–3.26	10	<3%	128	1.6 V/–1.6 V@ 50 ns
TiN/PCMO/Pt[33]	Interfacial	–3.4/–2.5	100	<2%	100	–1.5~ –3.5 V /0.5~2.5 V @ 100 μs
Pt/AlO_xN-rich TiN/PCMO/Pt[34]	Interfacial	3.68/–6.76	6.8	<1%	50	3 V/–3 V @ 1 ms
TiN/HZO/SiO_2/Si FeFET[35]	FeFET	1.75/1.46	45	<0.5%	32	2.85~4.45 V/–2.1~ –3.8 V @75 ns
TiN/HZO/SiO_2/Si FeFET[35]	FeFET	5.54/–8.08	8	–	20	3.7 V/–3.2 V@75 ns
Pt/BTO/Nb:STO[36]	FTJ	–	10	2.5%	200	1.3 V/–1.75 V@50 ns
Au/PVDF/Nb:STO[37]	FTJ	1.2/1.6	2.2	–	75	2 V/–7 V@ 20 ns
Our work	FTJ	0.77/–0.94	100	2.06%	256	1.35~2 V/–1.4~ –3.5 V@ 10 ns
		1.20/–1.09	30	3.65%	150	3.5~4.5 V/–3.2~ –5 V@ 630 ps

S12. Neural network simulation for online supervised learning

Online supervised learning was performed based on the ResNet-18 convolutional neural network (CNN) to recognize 28×28 pixel images in Fashion-MNIST by pytorch. To verify the noise tolerance of the neural network, images with salt & pepper noise or Gaussian noise were generated and recognized. For salt & pepper noise, the grayscales of some randomly chosen pixels were set to be white or black, and the ratio of chosen pixel number to the total pixel number β is defined as noise level. While for Gaussian noise, noises that obey a Gaussian distribution with zero mean and standard deviation values ζ (ratio to the maximum pixel intensity, defined as noise level) were added to the images[38,39].

Neural network construction. The ResNet-18 neural network contains 17 convolution layers and a final full connection layer, as shown in Fig. 7a of the main text. For the first convolution layer, the size of convolution kernel is 5×5. Afterwards, there are 8 blocks, and each block contains 2 convolutional layers. The sizes of convolution kernels for these blocks are all 3×3. The number of kernels increases from 64 for B1, 128 for B2, 256 for B3, to 512 for B4 block. And the feature size decreases from 32 for B1, 16 for B2, 8 for B3, to 4 for B4 block. The input image data converted from each pixel grayscale will flow through each convolution block by two ways simultaneously. In one way, the data are convolved and flow through the convolution block. In the other way, the data are transmitted around the convolution block by a shortcut connection. These two types of data are summed after each block and transmitted through blocks one by one. It is noted that there are two kinds of shortcut connections. In the shortcut connections for B1_1, B1_2, B2_2, B3_2 and B4_2 convolutional layers, data are transmitted directly and are plotted as solid arrows in Fig. 7a. While in the shortcut connections for B2_1, B3_1 and B4_1 convolutional layers, data are convolved by 1×1 convolution kernels and are plotted as dotted arrows in Fig. 7a. The last convolutional block B4_2 is followed by the final full connection layer with 10 output neurons.

Neural network training and testing. The training of the neural network is composed of two main steps: feedforward inference and feedback weight updates. During the inference process, the grayscale value in each pixel of an image was encoded to a read voltage amplitude. The read voltage vectors were input to top electrodes of synaptic devices, and the recognition result is based on the current from bottom electrode. For each training cycle, 1024 images were selected from F-MNIST training dataset as a batch. In each cycle, the multilayer inference was performed layer by layer. During the feedback

weight update process, the desired weight update is calculated based on stochastic gradient descent (SGD) and back propagation (BP) algorithms[31]. Based on the experimental results in Fig. 6 of the main text, the mean value and cycle-to-cycle standard deviation of each conductance state can be obtained, as shown in Supplementary Fig. S11, and these results were used as the device behavioral model for the neural network simulations[40]. The cycle-to-cycle variations were added as random fluctuations following Gaussian distribution in certain ranges.

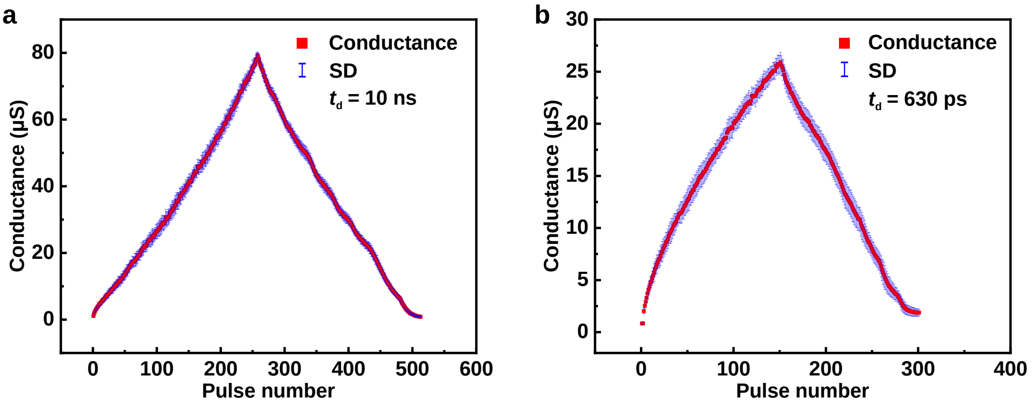

Supplementary Fig. S11 Device behavioral model. a, b Mean value and standard deviation of conductance *vs.* pulse number with pulse durations of t_d = 10 ns and t_d = 630 ps, respectively. The error bars indicate the standard deviations (SD) of each conductance state, representing the cycle-to-cycle variations.

Table S4 Conductance manipulation properties and simulation results

	Based on 630 ps data	Based on 10 ns data
Number of states	150	256
Cycle-to-cycle variation	3.65%	2.06%
Nonlinearity	1.20 (LTP), –1.09 (LTD)	0.77 (LTP), –0.94 (LTD)
Accuracy for **F-MNIST**	90.0%	94.7%
Accuracy for **MNIST**	99.1%	99.5%

Under the pulsed voltages with a duration of 10 ns, the FTJs show high performance in conductance manipulations with multiple states (256), small cycle-to-cycle variation (2.06%) and linear conductance manipulation (nonlinearity <1). Thus, the network based on these parameters shows a high recognition accuracy of 94.7% for clear F-MNIST images (see Table S4), which is close to that of 95.6% achieved by floating-point-based software. When the CNN was simulated based on the 150 states with 630 ps pulse duration,

the recognition accuracy is degraded (~90.0%), due to the relatively higher cycle-to-cycle variation, lower linearity and less conductance states. While for F-MNIST images with noises, the recognition accuracies maintain >90% when salt & pepper noise level is 0.3 and Gaussian noise level is 0.2, as shown in Fig. 7e.

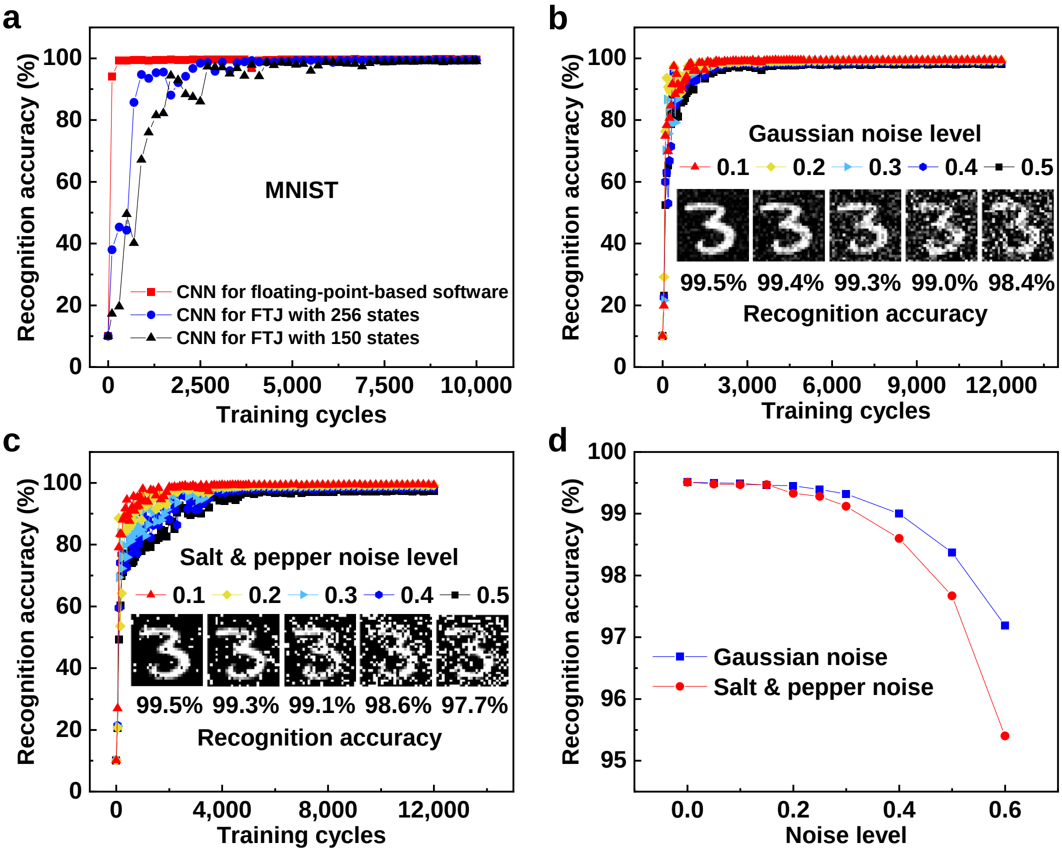

Supplementary Fig. S12 CNN simulation for MNIST images. a Simulation results based on the experimental results with 256 (in Fig. 6a of main text) and 150 (in Fig. 6b) conductance states and floating-point-based software for clear MNIST images. **b, c** Training results of noisy MNIST images with different levels of Gaussian noise and salt & pepper noise, respectively. **d** Recognition accuracy of noisy MNIST images with different levels of Gaussian noise and salt & pepper noise.

In addition, the CNN simulations on common MNIST dataset were also carried out. As shown in Fig. S12a, for clear MNIST images, high recognition accuracies of 99.7%, 99.5% and 99.1% were achieved in the CNN simulations based on floating point, 256 states and 150 states, respectively. For noisy patterns, the recognition can still be >99% when salt & pepper noise is 0.3 and Gaussian noise is 0.4.

S13. Ultrafast test circuit with a waveguide

To carry out ultrafast measurements, an ultrafast test circuit is carefully designed with the FTJ connected to the circuit through a microstrip waveguide[41,42]. The characteristic impedance Z_0 = 50 Ω of the designed microstrip waveguide matches with the connection cables and instruments (see Methods of manuscript). To characterize the transmission capability, the microstrip waveguide was used to test a short copper wire by using a vector network analyzer (AV3656A, CETC-41, China), as the transmission and reflection properties up to 3 GHz shown in Fig. S13a and b. It can be seen that the magnitude of transmission through the waveguide is high (≥–2.2 dB) and the shift of transmitted phase varies linearly with frequency, indicating the weak dispersion which ensures the pulse integrity[22].

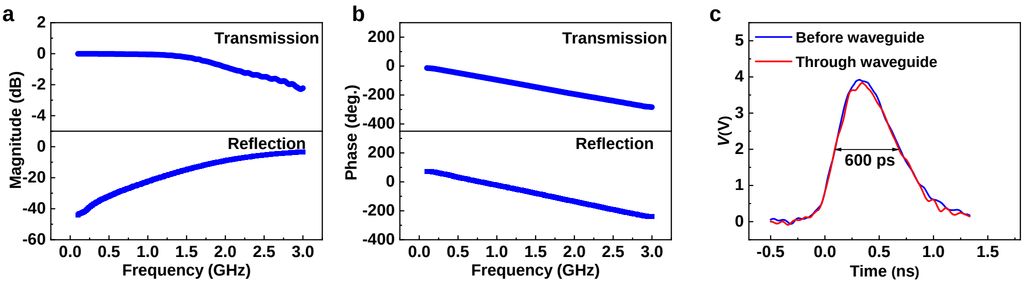

Supplementary Fig. S13 High frequency characterizations of microstrip waveguide. **a** Magnitudes and **b** phases of transmission and reflection. **c** The ~600 ps waveforms before and through the microstrip waveguide.

The pulsed voltages before and after passing through the waveguide were measured by an oscilloscope, as shown in Fig. S13c. The pulse ~600 ps shows little shape deformation after transmitted through the waveguide, which implies that the voltage pulse maintains its shape when it is delivered to the device.

The circuit was further characterized after connecting the FTJ onto the waveguide by short copper wires. High frequency scattering (S)-parameter measurements from 0.1 to 3 GHz were performed, as shown in Fig. S14a-d. Here, for the phase data in Fid. S14b, d, the phase shift contributed from the microstrip lines has been subtracted[22]. An equivalent circuit model is used to analyze the results, similar to the previous work[22]. As shown in Fig. S14e, C_m is the parasitic capacitance of FTJ, R_s is the series contact resistance of the FTJ and waveguide, and R_m is the parallel resistance that is approximately equal to the FTJ resistance. Similar to the earlier reports[22,43], the S-parameters of transmission (V_{trans}/V_{inc}) and reflection (V_{refl}/V_{inc}) can be calculated by Equations S8 and S9, respectively:

$$\frac{V_{trans}}{V_{inc}} = \left(\frac{2Z_0}{2Z_0+Z_L}\right), \tag{S8}$$

$$\frac{V_{\mathrm{refl}}}{V_{\mathrm{inc}}} = \left(\frac{Z_L}{2Z_0+Z_L}\right), \tag{S9}$$

where Z_0 = 50 Ω is the characteristic impedance and Z_L is the impedance of the equivalent circuit that was calculated by advanced design system (ADS) software. The results in Fig. S14a-d can be described nicely by the equivalent circuit model simulated using ADS software. For the OFF state, the R_s is ~36 Ω, C_m is ~4.8 pF, and R_m is approximately equal to 10^6 Ω. Thus, the RC delay can be estimated to be $R_s \times C_m$ = 172 ps[23]. While for the ON state, the R_s is ~30 Ω, C_m is ~5.1 pF, R_m is approximately equal to 10^4 Ω, and thus the RC delay is estimated to be 153 ps. It is noted that the RC delays are shorter than the widths of applied pulses, showing that subnanosecond pulse signal can be applied to the FTJ devices successfully.

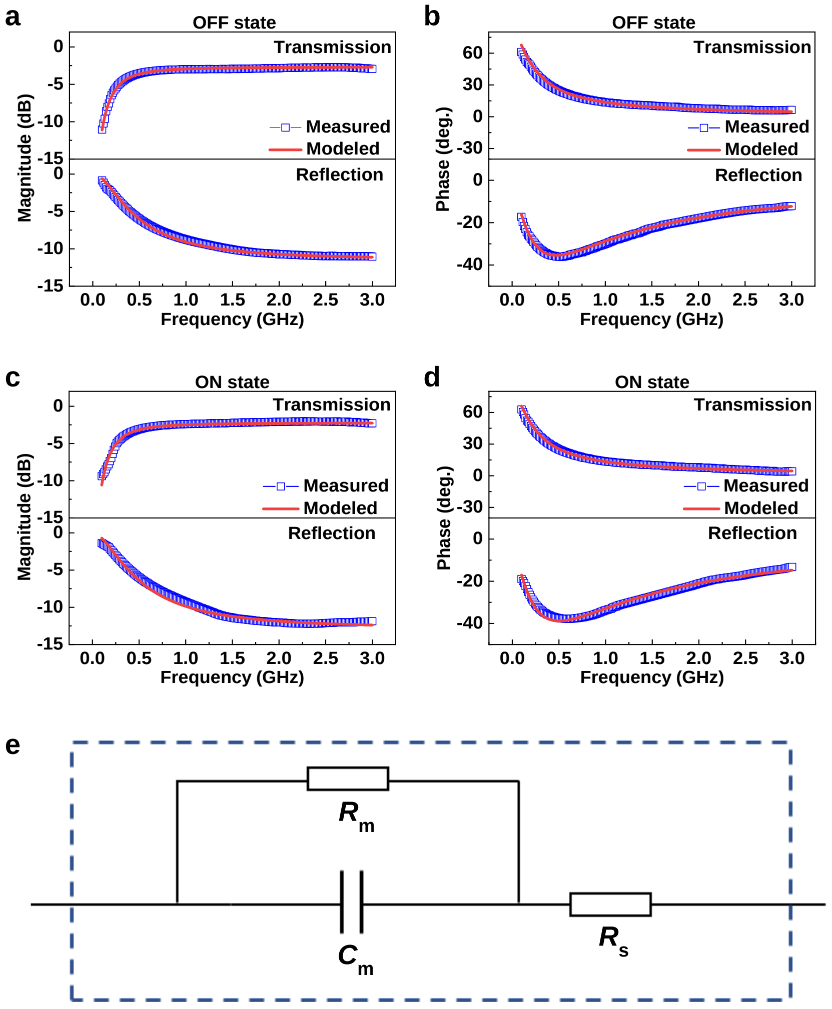

Supplementary Fig. S14 S-parameter characterized by a vector network analyzer. a Magnitudes and **b** phases of transmission and reflection of the FTJ measured at OFF state, **c** magnitudes and **d** phases of reflection and transmission of the FTJ measured at ON state. **e** The equivalent circuit model used for the simulations.

Supplementary References

1 Yu, S. M. Neuro-inspired computing with emerging nonvolatile memorys. *Proc. IEEE* **106**, 260-285 (2018).

2 Zhang, T., Yang, K., Xu, X. Y., Cai, Y. M., Yang, Y. C. & Huang, R. Memristive devices and networks for brain-inspired computing. *Phys. Status Solidi RRL* **13**, 1900029 (2019).

3 Zhang, W. Q., Gao, B., Tang, J. S., Yao, P., Yu, S. M., Chang, M. F., Yoo, H. J., Qian, H. & Wu, H. Q. Neuro-inspired computing chips. *Nat. Electron.* **3**, 371-382 (2020).

4 Upadhyay, N. K., Jiang, H., Wang, Z. R., Asapu, S., Xia, Q. F. & Yang, J. J. Emerging memory devices for neuromorphic computing. *Adv. Mater. Technol.* **4**, 1800589 (2019).

5 Padovani, F. A. & Stratton, R. Field and thermionic-field emission in Schottky barriers. *Solid-State Electron.* **9**, 695-707 (1966).

6 Xi, Z. N., Ruan, J. J., Li, C., Zheng, C. Y., Wen, Z., Dai, J. Y., Li, A. D. & Wu, D. Giant tunnelling electroresistance in metal/ferroelectric/semiconductor tunnel junctions by engineering the Schottky barrier. *Nat. Commun.* **8**, 15217 (2017).

7 Barrett, J. H. Dielectric constant in perovskite type crystals. *Phys. Rev.* **86**, 118 (1952).

8 Susaki, T., Kozuka, Y., Tateyama, Y. & Hwang, H. Y. Temperature-dependent polarity reversal in Au/Nb:$SrTiO_3$ Schottky junctions. *Phys. Rev. B* **76**, 155110 (2007).

9 Scott, J. F., Watanabe, K., Hartmann, A. J. & Lamb, R. N. Device models for PZT/Pt, BST/Pt, SBT/Pt, and SBT/Bi ferroelectric memories. *Ferroelectrics* **225**, 83-90 (1999).

10 Guo, D. Y., Liu, H., Li, P. G., Wu, Z. P., Wang, S. L., Cui, C., Li, C. R. & Tang, W. H. Zero-power-consumption solar-blind photodetector based on β-Ga_2O_3/NSTO heterojunction. *ACS Appl. Mater. Interfaces* **9**, 1619-1628 (2017).

11 Kay, H. F. & Dunn, J. W. Thickness dependence of the nucleation field of triglycine sulphate. *Philos. Mag.* **7**, 2027-2034 (1962).

12 Mimura, T., Shimizu, T., Uchida, H., Sakata, O. & Funakubo, H. Thickness-dependent crystal structure and electric properties of epitaxial ferroelectric Y_2O_3-HfO_2 films. *Appl. Phys. Lett.* **113**, 102901 (2018).

13 Steffes, J. J., Ristau, R. A., Ramesh, R. & Huey, B. D. Thickness scaling of ferroelectricity in $BiFeO_3$ by tomographic atomic force microscopy. *Proc. Natl. Acad. Sci. U.S.A.* **116**, 2413-2418 (2019).

14 Li, C. J., Huang, L. S., Li, T., Lv, W. M., Qiu, X. P., Huang, Z., Liu, Z. Q., Zeng, S. W., Guo, R., Zhao, Y. L., Zeng, K. Y., Coey, M., Chen, J. S., Ariando, & Venkatesan, T. Ultrathin $BaTiO_3$-based ferroelectric tunnel junctions through interface engineering. *Nano Lett.* **15**, 2568-2573 (2015).

15 Xi, Y., Gao, B., Tang, J. S., Chen, A., Chang, M. F., Hu, X. B. S., Van Der Spiegel, J., Qian, H. & Wu, H. Q. In-memory learning with analog resistive switching memory: a review and perspective. *Proc. IEEE* **109**, 14-42 (2021).

16 Wang, Z. R., Joshi, S., Savel'ev, S. E., Jiang, H., Midya, R., Lin, P., Hu, M., Ge, N., Strachan, J. P., Li, Z. Y., Wu, Q., Barnell, M., Li, G. L., Xin H. L., Williams, R. S., Xia, Q. F. & Yang, J. J. Memristors with diffusive dynamics as synaptic emulators for neuromorphic computing. *Nat. Mater.* **16**, 101-108 (2017).

17 Yoon, J. H., Wang, Z. R., Kim, K. M., Wu, H. Q., Ravichandran, V., Xia, Q. F., Hwang, C. S. & Yang, J. J. An artificial nociceptor based on a diffusive memristor. *Nat. Commun.* **9**, 417 (2018).

18 Hernandez-Martin, D., Gallego, F., Tornos, J., Rouco, V., Beltran, J. I., Munuera, C., Sanchez-Manzano, D., Cabero, M., Cuellar, F., Arias, D., Sanchez-Santolino, G., Mompean, F. J., Garcia-Hernandez, M., Rivera-Calzada, A., Pennycook, S. J., Varela, M., Muñoz, M. C., Sefrioui, Z., Leon, C. & Santamaria, J. Controlled sign reversal of electroresistance in oxide tunnel junctions by electrochemical-ferroelectric coupling. *Phys. Rev. Lett.* **125**, 266802 (2020).

19 Boyn, S., Grollier, J., Lecerf, G., Xu, B., Locatelli, N., Fusil, S., Girod, S., Carrétéro, C., Garcia, K., Xavier, S., Tomas, J., Bellaiche, L., Bibes, M., Barthélémy, A., Saïghi, S. & Garcia, V. Learning through ferroelectric domain dynamics in solid-state synapses. *Nat. Commun.* **8**, 14736 (2017).

20 Boyn, S., Chanthbouala, A., Girod, S., Carrétéro, C., Barthélémy, A., Bibes, M., Grollier, J., Fusil, S. & Garcia, V. Real-time switching dynamics of ferroelectric tunnel junctions under single-shot voltage pulses. *Appl. Phys. Lett.* **113**, 232902 (2018).

21 Ma, C., Luo, Z., Huang, W. C., Zhao, L. T., Chen, Q. L., Lin, Y., Liu, X., Chen, Z. W., Liu, C. C., Sun, H. Y., Jin, X., Yin, Y. W. & Li, X. G. Sub-nanosecond memristor based on ferroelectric tunnel junction. *Nat. Commun.* **11**, 1439 (2020).

22 Torrezan, A. C., Strachan, J. P., Medeiros-Ribeiro, G. & Williams, R. S. Sub-nanosecond switching of a tantalum oxide memristor. *Nanotechnology* **22**, 485203 (2011).

23 Wang, C., Wu, H. Q., Gao, B., Wu, W., Dai, L. J., Li, X. Y. & Qian, H. Ultrafast RESET analysis of HfO_x-based RRAM by sub-nanosecond pulses. *Adv. Electron. Mater.* **3**, 1700263 (2017).

24 Cao, Y., Rushforth, A. W., Sheng, Y., Zheng, H. Z. & Wang, K. Y. Tuning a binary ferromagnet into a multistate synapse with spin–orbit-torque-induced plasticity. *Adv. Funct. Mater.* **29**, 1808104 (2019).

25 Ding, K. Y., Wang, J. J., Zhou, Y. X., Tian, H., Lu, L., Mazzarello, R., Jia, C. L., Zhang, W., Rao, F. & Ma, E. Phase-change heterostructure enables ultralow noise and drift for memory operation. *Science* **366**, 210-215 (2019).

26 Wang, C. C., Hou, Z. Y., Deng, Y. L., Chio, U. F., & Wang, W. 2-GHz 2×VDD 28-nm CMOS digital output buffer with slew rate auto-adjustment against processand voltage variations. *J. Circuits, Syst. Comput.* **29**, 2050088 (2020).

27 Mielke, N. R., Frickey, R. E., Kalastirsky, I., Quan, M. Y., Ustinov, D. & Vasudevan, V. J. Reliability of solid-state drives based on nand flash memory. *Proc. IEEE* **105**, 1725-1750 (2017).

28 Kim, S. W., Yang, J. H., Park, E. J., Choi, J. M. & Kwon, K. W. A high efficiency variable stage and frequency charge pump for wide range ISPP. *IEEE International Symposium on Circuits and Systems (ISCAS)*, 1-5 (2020).

29 Park, M. H., Lee, Y. H., Mikolajick, T., Schroeder, U. & Hwang, C. S. Review and perspective on ferroelectric HfO_2-based thin films for memory applications. *MRS Commun.* **8**, 795-808 (2018).

30 Yamaguchi, T., Zhang, T., Omori, K., Shimada, Y., Kunimune, Y., Ide, T., Inoue, M. & Matsuura, M. Highly reliable ferroelectric $Hf_{0.5}Zr_{0.5}O_2$ film with Al nanoclusters embedded by novel sub-monolayer doping technique. *International Electron Devices Meeting (IEDM)* 7.5.1-7.5.4 (IEEE, San Francisco, CA, USA, 2018).

31 Li, C., Belkin, D., Li, Y. N., Yan, P., Hu, M., Ge, N., Jiang, H., Montgomery, E., Lin, P., Wang, Z. R., Song, W. H., Strachan, J. P., Barnell, M., Wu, Q., Williams, R. S., Yang, J. J. & Xia, Q. F. Efficient and self-adaptive in-situ learning in multilayer memristor neural networks. *Nat. Commun.* **9**, 2385 (2018).

32 Wu, W., Wu, H. Q., Gao, B., Deng, N., Yu, S. M. & Qian, H. Improving analog switching in HfO_x-based resistive memory with a thermal enhanced layer. *IEEE Electron. Device Lett.* **38**, 1019-1022 (2017).

33 Jang, J. W., Park, S. S., Burr, G. W., Hwang, H. S. & Jeong, Y. H. Optimization of conductance change in $Pr_{1-x}Ca_xMnO_3$-based synaptic devices for neuromorphic systems. *IEEE Electron. Device Lett.* **36**, 457-459 (2015).

34 Park, S., Sheri, A., Kim, J., Noh, J., Jang, J., Jeon, M., Lee., B., Lee, B. R., Lee, B. H. & Hwang, H. Neuromorphic speech systems using advanced ReRAM-based synapse. *International Electron Devices Meeting (IEDM)* 25.6.1-25.6.4 (IEEE, Washington, DC, USA, 2013).

35 Jerry, M., Chen, P. Y., Zhang, J. C., Sharma, P., Ni, K., Yu, S. M. & Datta, S. Ferroelectric FET analog synapse for acceleration of deep neural network training *International Electron Devices Meeting (IEDM)* 6.2.1-6.2.4 (IEEE, San Francisco, CA, USA, 2017).

36 Li, J. K., Ge, C., Du, J. Y., Wang, C., Yang, G. Z. & Jin, K. J. Reproducible ultrathin ferroelectric domain switching for high-performance neuromorphic computing. *Adv. Mater.* **32**, 1905764 (2020).

37 Majumdar, S. Ultrafast switching and linear conductance modulation in ferroelectric tunnel junctions via P(VDF-TrFE) morphology control. *Nanoscale* **13**, 11270-11278 (2021).

38 Chen, J., Pan, W. Q., Li, Y., Kuang, R., He, Y. H., Lin, C. Y., Duan, N., Feng, G. R., Zheng, H. X., Chang, T. C., Sze, S. M. & Miao, X. S. High-precision symmetric weight update of memristor by gate voltage ramping method for convolutional neural network accelerator. *IEEE Electron Device Lett.* **41**, 353-356 (2020).

39 Kim, H. J., Hwang, S. M., Park, J. J., Yun, S. D., Lee, J. H. & Park, B. G. Spiking neural network using synaptic transistors and neuron circuits for pattern recognition with noisy images. *IEEE Electron Device Lett.* **39**, 630-633 (2018).

40 Prezioso, M., Merrikh-Bayat, F., Hoskins, B. D., Adam, G. C., Likharev, K. K. & Strukov, D. B. Training and operation of an integrated neuromorphic network based on metal-oxide memristors. *Nature* **521**, 61-64 (2015).

41 Pozar, D. M. *Microwave Engineering (4th edition)*. John wiley & sons, (2012).

42 Choi, B. J., Torrezan, A. C., Strachan, J. P., Kotula, P. G., Lohn, A. J., Marinella, M. J., Li, Z. Y., Willians, R. S. & Yang, J. J. High-speed and low-energy nitride memristors. *Adv. Funct. Mater.* **26**, 5290-5296 (2016).

43 Abuwasib, M., Lee, H., Lee, J. W., Eom, C. B., Gruverman, A. & Singisetti, U. Characterization and modeling of Co/$BaTiO_3$/$SrRuO_3$ ferroelectric tunnel junction memory by capacitance–voltage (C–V), current–voltage (I–V), and high frequency measurements. *IEEE Trans. Electron. Devices* **66**, 2186-2191 (2019).
